# Successful expectant management of tubal heterotopic pregnancy

**DOI:** 10.4103/0974-1208.69333

**Published:** 2010

**Authors:** Asha Baxi, Manila Kaushal, HK Karmalkar, Peeti Sahu, Pooja Kadhi, Baxi Daval

**Affiliations:** Disha Fertility and Surgical Center, Indore, (M. P.), India; 1 Consultant Radiologist, Medicare Center, Indore, India

**Keywords:** Expectant management, heterotopic pregnancy, pelvic inflammatory disease

## Abstract

Expectant management for tubal heterotopic pregnancy could be considered as a successful option in a symptom-free patient where the ectopic embryo has a limited craniocaudal length with no cardiac activity. We report the obstetric outcome after expectant management for a right tubal heterotopic pregnancy. Heterotopic pregnancy was first recognized at 6 weeks gestation in a 32-year-old salpingectomized woman with an 8-year history of subfertility who conceived after *in utero* transfer of three embryos obtained by *in vitro* fertilization. Expectant management and close ultrasonographic and clinical monitoring were done. The intrauterine pregnancy proceeded unremarkably. A cesarean section was performed for breech presentation, and it allowed the delivery of a healthy 2260-g male infant. The examination of the adnexa showed a pre-rupture of the right fallopian tube.

## INTRODUCTION

Heterotopic pregnancy, a rare phenomenon in the past, is now becoming more common because of assisted reproductive technique. Heterotopic pregnancies have increased alongside the advent of assisted reproductive technique.[1] Ectopic pregnancy is a gynecologic emergency, generally requiring expeditious surgical or medical treatment. However, in a small number of cases in which the risk of tubal rupture is minimal, expectant management is appropriate. In patients in whom the diagnosis of ectopic pregnancy can be made without laparoscopy and who sonographically demonstrate an un-ruptured gestation and a persistent downward trend to the beta-human chorionic gonadotrophin (beta-hCG) assay, expectant management has been successfully applied.[2,3] This form of conservative management can also be applied to heterotopic pregnancies having a similar clinical appearance. In expectant management, no treatment is given and the patient is followed closely with weekly transvaginal ultrasonography.[4] We report a case of successful expectant management of tubal heterotopic pregnancy.

## CASE REPORT

A 32-year-old woman came for treatment of infertility in November 2005. She gave history of ectopic pregnancy on two occasions. First ectopic pregnancy occurred in left tube, which was treated with laparoscopic salpingectomy in the year 1999. Again she had ruptured ectopic pregnancy a year later in the same tube, which was managed by laparoscopic salpingectomy. She underwent hysterosalpingography, which showed block in the right fallopian tube. She had past history of abdominal Koch’s (tuberculosis), for which she had received anti-tubercular treatment for 9 months. She had one attempt of *In Vitro* Fertilization-Embryo Transfer (IVF-ET) in 2002, which had failed, and this was her second attempt. Three embryos were transferred; and 14 days later, the beta-hCG was 228 mIU/mL. A transvaginal scan at 6 weeks revealed one intrauterine gestational sac with fetal pole and fetal heart. There was also an extrauterine sac measuring 1.6 cm in right adnexal region with live fetus. This was confirmed by two different sonologists. The couple was explained about the severity of the situation, and the option to choose between laparoscopic salpingectomy and ultrasound-guided intra-cardiac instillations of potassium chloride (KCL) was offered. However the couple deferred for any form of treatment as she was a asymptomatic. We decided to keep her under observation by serial ultrasound scans and clinical examination.

After 1 week, transvaginal scan revealed one intrauterine sac of 6 weeks 6 days measuring 1.8 cm with tiny fetal pole and gestational sac measuring 1.75 cm in right adnexa. There was no significant free fluid in pouch of Doughlas (POD). Patient complained of constant niggling, pain but her general condition was good. Her vitals and hematological parameters were normal and therefore, expectant management was continued.

Another ultrasound a week later revealed continuing live intrauterine pregnancy of 8 weeks 3 days gestation, with mild sac separation. Adnexal mass had increased but without cardiac activity and with no free fluid in POD.

Further serial ultrasound examinations showed a growth of the intrauterine gestation and regression of the ectopic pregnancy, which completely regressed by 13 weeks. The patient presented with premature rupture of membranes at 36 weeks of gestation and breech presentation. A lower segment cesarean section was done on September 1, 2006, and a healthy male child weighing 2260 g was delivered. At cesarean section, both ovaries were normal, right fallopian tube was empty and hyperemic while left tube was absent.

## DISCUSSION

Heterotopic pregnancy is a diagnostic masquerader. A universal characteristic of a good “early diagnostic” protocol is a “high index of suspicion.” Spontaneous heterotopic pregnancy is a rare event and the incidence is 1: 30,000 pregnancies.[2] As more and more infertile couples turn to assisted reproductive technique, the incidence of heterotopic pregnancy has expectedly increased from 1.9% to 2.9%.[5] If the patient has had history of previous pelvic inflammatory disease or tubal pathology, there will be an obvious increase in rate of occurence of pregnancies.[7] In our case, the patient had previous history of tuberculosis, pelvic adhesions and transfer of multiple embryos during IVF — all of which are considered risk factors of heterotopic pregnancy.

The most important aid in the diagnosis of heterotopic pregnancy is the utilization of high-resolution transvaginal ultrasonography.[6,7] In high-risk patients, especially those who have conceived with assisted reproductive technique, a routine ultrasound scanning for ectopic or heterotopic pregnancy at 4 and 6 weeks after transfer of embryos is recommended. On the other hand, abdominal pain, rebound tenderness, fluid in the POD at trans-vaginal scan (TVS) examination and a low serum hemoglobin percentage were independent predictors of tubal ruptures or active bleeding. In our case, the patient was asymptomatic, and heterotopic pregnancy was diagnosed on a routine transvaginal ultrasound.

The management of heterotopic pregnancy still remains controversial. Operative management is still a mainstay, but it involves surgical and anesthetic risk to both the mother and fetus.[8] Although it has been reported that laparotomy does not seem to interrupt intrauterine pregnancy,[9] others have reported a 40% loss of viable fetuses.[10] Several others have mentioned the value and safety of laparoscopy in the diagnosis and treatment.[11] Methotrexate with its potential adverse effects on the intrauterine gestation and RU486 a prostaglandins, with their potential effect on uterine contractility, are not options in the treatment of ongoing heterotopic pregnancy. The injection of potassium chloride to selectively reduce multiple intrauterine gestations has been widely used. More recently this approach has been used to manage heterotopic pregnancy. Wright *et al*. reported a case of selective embryo reduction of the tubal gestation of a heterotopic pregnancy resulting from IVF-embryo transfer, but this resulted in a hematosalpinx requiring mini-laparotomy and salpingectomy.[12]

So far, few cases of expectant management of heterotopic pregnancy have been reported.[3,13–15] In our case, as the patient was asymptomatic and had opted for expectant management with regular follow-up despite being informed of possible complications. Adequate counselling and judicious follow-up of the patient are the essential components of expectant management. The use of more stringent selection criteria results in increase in the efficacy of expectant management to approximately 70%.[2] Success rates vary from 48% to 100%, depending on differences in inclusion criteria adopted.[16]

## CONCLUSION

Expectant management can be a useful form of treatment for heterotopic pregnancy in selected cases; however, we are not recommending expectant management as a routine treatment. A substantially favorable risk-to-benefit ratio should be demonstrated before the adoption of expectant management.
